# Flour a Remedy for Burns and Scalds

**Published:** 1829

**Authors:** 


32. *Flour a remedy for Burns and Scalds.*—Mr. Marshall, an English Surgeon, lately published in the London Medical and Physical Journal, the minutes of several cases of burns treated with flour. We shall transcribe the following as a favorable specimen of the whole.

A boy scalded the left ankle-joint and upper part of the foot. He had applied Goulard's lotion the first two days; and afterwards a dressing, spread on lint, of red precipitate rubbed down with yellow basilcon. Five days after the accident I saw the patient; the part was highly inflamed, and nearly covered with blisters, which had been injudiciously opened, in a state of rapidly spreading ulceration, with a purulent discharge. He could neither use the joint nor bend the toes, being stiff from painful distension. The stimulating dressing was carefully wiped off, where practicable, and the flour substituted. The youth expressed immediate relief. He was directed to apply it every hour during the day, and as often as he awoke in the night, and, wherever the discharge issued through the layer, to apply the flour more assiduously.

Second day.—The patient had passed a good night. Swelling nearly subsided; the surrounding inflammation gone; the ulcers mostly healed; one of them contained a portion of fluid, and another, near the inner ankle, gave out a discharge of matter. He moved the toes and ankle-joint freely. The change was very remarkable.

Third day.—The fluctuating matter that appeared on the preceding day was wholly absorbed. Patient free from pain. The ulcer near the inner ankle gave off a trifling discharge. On removing from the surface the coat of flour to inspect the character of the granulation, it was found in a most healthy and healing condition. The frightful aspect of a general, ill-conditioned, and irritable ulcer, which threatened mischief on the first view, was effectually removed.

Fourth day.—The surface of the part affected was washed in tepid water, in order to obtain a full view of its state. The whole was healed, except two small places, not so large as a horsebean, which were in a healthy healing condition. The new skin was of a red hue, and shining.

On the *modus curandi* of this new treatment, he has the following observations:

This mild substance is doubtless pre-eminent to all others hitherto in use, by imparting immediate ease to the inflamed and irritable surface; it rapidly heals by the scabbing process, in uniting with the discharge from the abraded cutis; and almost instantaneously forms a temporary semi-transparent covering, thereby assisting the natural functions in restoring the epidermis. The advantage becomes evident by stopping a profuse discharge, and the tedious progress of ulceration. That remarkable substance, the animal gluten, peculiarly contained in wheat, seems in this instance to assist the rapid regeneration of the scarfskin, and thus protects the cutis and rete mucosum. The surfuce of the body being wonderfully supplied by the extension of the cutaneous nerves in the form of a soft pulpy membrane, somewhat resembling the expansion of the optic nerve on the retina, readily affords, it is presumed, an explanation of the great violence offered to the system in all cases of extensive burn or scald.

When the flour has formed the artificial covering, the further application becomes comparatively superfluous; which is perceived by its rolling off.

